# Quasi-Complete Response of Classic Kaposi's Sarcoma Treated with Weekly Paclitaxel

**DOI:** 10.1155/2013/196878

**Published:** 2013-02-14

**Authors:** Zineb Benbrahim, Samia Arifi, Hafida Benhammane, Kaoutar Inani, Salim Gallouj, Meriem Meziane, Fatima Zahra Mernissi, Nawfel Mellas, Omar El Mesbahi

**Affiliations:** ^1^Medical Oncology, Hassan II University Hospital, P.O. Box 8743, Fès, Morocco; ^2^Service of Dermatology, Hassan II University Hospital, Fès, Morocco

## Abstract

Classic Kaposi's sarcoma (CKS) is a subtype that traditionally occurs in elderly HIV-negative males of Mediterranean origin. Patients with CKS characteristically present with skin lesions in the distal extremities. Involvement of the viscera is uncommon but may occur in the late stages of the disease. Patients with extensive KS can be treated with systemic chemotherapy. A number of drugs approved for treatment of AIDS-associated KS, especially Paclitaxel, have activity against CKS after failure of prior therapy. We report a patient treated with weekly Paclitaxel, as initial chemotherapy, for CKS presenting with multiple visceral involvement and having a contraindication for Bleomycin. The patient had quasi-complete response after three months of chemotherapy suggesting that weekly Paclitaxel might be effective as a first-line therapy for classical type KS with visceral involvement.

## 1. Introduction

Classic Kaposi's sarcoma (CKS) is an angioproliferative disorder that is thought to develop from endothelial cells, myofibroblasts, and monocyte-macrophages. Widespread, or rapidly progressive CKS, is an indication for systemic chemotherapy. Although no cytotoxic chemotherapeutic agents have been approved for treatment of CKS, a number of drugs indicated for AIDS-associated KS have activity against CKS. These include Bleomycin, Vinblastine, Etoposide, Pegylated liposomal Doxorubicin, Gemcitabine, and Paclitaxel [1–5]. We report a case of a major response after 3 months of weekly Paclitaxel treatment for multiple visceral localizations of classic Kaposi's sarcoma.

## 2. Case Report

**Figure 1:**
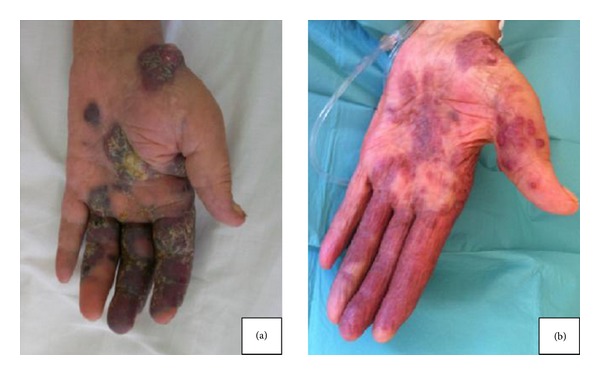
(a) Hand lesions of classic Kaposi's sarcoma. (b) Evolution of the lesions after treatment by Paclitaxel.

A 80-year-old male was admitted to Hassan II University Hospital in 2010 for treatment of long-standing Kaposi's sarcoma. Disease had presented 2 years earlier with the appearance of a few papules on his legs with gradual extension. Physical examination showed multiple violaceous to dark red patches and plaques of varying sizes on his face, trunk, and limbs with acral predominance (Figure 1(a)). Similar lesions were observed in the buccal and genital mucosa. There was severe bilateral lymphedema of the legs. A biopsy specimen from a typical lesion revealed vague areas of interlacing fascicles of spindle cells associated with patchy infiltration by lymphocytes and hemorrhagic suffusions confirming the clinical diagnosis of KS. Human immunodeficiency virus (HIV) antibody was negative. Thoraco-abdomino-pelvic scans showed multiple lesions in the right lung, pleural effusion, mediastinal lymphadenopathy, and suprarenal and splenic lesions. On esophagogastroduodenoscopy, a polypoid reddish mucosal lesion was found in the fundus of the stomach. Histological examination of this fundic lesion revealed gastric involvement by Kaposi's sarcoma. We prescribed systemic chemotherapy including Bleomycin. However, Plethysmography revealed a restrictive pulmonary syndrome contraindicating Bleomycin. The patient was treated with Paclitaxel 100 mg weekly. There were no side effects reported. After three months of chemotherapy, we observed a major decrease in leg lesions and edema with remarkable improvement of cutaneous lesions and only residual scarring over the insteps remained (Figure 1(b)). The CT scan showed disappearance of the suprarenal and splenic lesions and marked decrease in mediastinal adenopathy and pulmonary lesions. The patient is currently in complete remission after one year of followup.

## 3. Discussion

Classic Kaposi's sarcoma is primarily a cutaneous disease of the lower extremities affecting predominantly elderly men of Mediterranean origin. This form is associated with an altered immune system and malignant diseases without HIV infection [6]. Clinically, this variant is characterized by multiple red to purple nodules on the lower limbs which grow larger. Histological feature shows submucosal vascular spindle cells with expression of CD 34, CD 31, and D2-40 by immunohistochemistry [7]. Involvement of internal organs occurs in approximately 10% of the classic form [8]. 

Patients with extensive or recurrent KS can be treated with systemic chemotherapy. A number of drugs approved for treatment of AIDS-associated KS have activity against CKS after failure of prior therapy. These include Vinblastine, Bleomycin, Doxorubicin, and Etoposide alone or in combination [1–4]. Paclitaxel has also shown efficacy with minimal toxicity in patients with classical KS after failure of prior therapy [5, 9]. In a recent report Fardet et al. describe 12 non-HIV-infected patients with refractory KS who were treated with Paclitaxel (175 mg/m^2^ every 3 wks) or Docetaxel (60 mg/m^2^ every 3 wks) [10]. For all patients, authors reported a partial response. The mean time to recurrence was 13 months. Toxicity was moderate. 

Recently, Brambilla et al. have evaluated the clinical efficacy and tolerability of Paclitaxel (100 mg weekly) in 17 patients with advanced aggressive and refractory CKS. The response to the therapy was evaluated after 12 weeks. Partial and/or complete response was achieved in 14 of 17 patients. The treatment was generally well tolerated. Mean time to recurrence was 4.5 months from the end of the therapy [11]. This study shows that low-dose Paclitaxel proved to be effective and well tolerated in patients with aggressive refractory CKS and can be repeated with good response.

In the case reported above, treatment was administered with low-dose Paclitaxel to minimize toxicity in an elderly patient with comorbidities and aggressive form of classic Kaposi's sarcoma. A major response and good tolerability were observed, suggesting that weekly Paclitaxel might be effective as first-line chemotherapy in treating classical type KS with visceral involvement.

## 4. Conclusion

The experience reported here would suggest that weekly low-dose Paclitaxel is effective as first-line chemotherapy in treating classical KS in life-threatening localizations. The low toxicity of this regimen is well tolerated, especially in elderly patients with comorbidities.
